# Practice effects persist over two decades of cognitive testing: Implications for longitudinal research

**DOI:** 10.1101/2025.06.16.25329587

**Published:** 2025-06-17

**Authors:** Jeremy A. Elman, Erik Buchholz, Rouhui Chen, Mark Sanderson-Cimino, Tyler R. Bell, Nathan Whitsel, Katherine J. Bangen, Alice Cronin-Golomb, Anders M. Dale, Lisa T. Eyler, Christine Fennema-Notestine, Nathan A. Gillespie, Eric L. Granholm, Daniel E. Gustavson, Donald J. Hagler, Richard L. Hauger, Diane M. Jacobs, Amy J. Jak, Mark W. Logue, Michael J. Lyons, Ruth E. McKenzie, Michael C. Neale, Robert E. Rissman, Chandra A. Reynolds, Rosemary Toomey, Arthur Wingfield, Hong Xian, Xin M. Tu, Carol E. Franz, William S. Kremen, Matthew S. Panizzon

**Affiliations:** a Department of Psychiatry, University of California San Diego, La Jolla, CA, USA; b Center for Behavior Genetics of Aging, University of California San Diego, La Jolla, CA, USA; c Department of Preventive Medicine, Feinberg School of Medicine, Northwestern University, Evanston, IL, USA; d Memory and Aging Center, Weill Institute for Neurosciences, San Francisco, CA, USA,; e VA San Diego Healthcare System, San Diego, CA, USA; f Department of Psychological and Brain Sciences, Boston University, Boston, MA, USA; g Department of Radiology, University of California San Diego, La Jolla, CA, USA; h Department of Neurosciences, University of California San Diego, La Jolla, CA, USA; i Virginia Institute for Psychiatric and Behavior Genetics, Richmond, Virginia, USA.; j Institute for Behavioral Genetics and Department of Psychology and Neuroscience, University of Colorado Boulder, Boulder, CO, USA,; k Center of Excellence for Stress and Mental Health (CESAMH), VA San Diego Healthcare System, San Diego, CA, USA; l Department of Psychiatry, Boston University School of Medicine, Boston, MA, USA.; m Biomedical Genetics, Boston University School of Medicine, Boston, MA, USA.; n Department of Biostatistics, Boston University School of Public Health, Boston, MA, USA.; o Department of Psychological and Brain Sciences, Boston University, Boston, MA, USA.; p Winston School of Education and Social Policy, Applied Human Development and Community Studies, Merrimack College, North Andover, MA, USA; q Department of Physiology and Neuroscience, Alzheimer’s Therapeutic Research Institute of the Keck School of Medicine of the University of Southern California, San Diego, CA, USA; r Department of Psychology and Volen National Center for Complex Systems, Brandeis University, Waltham, MA, USA; s Department of Epidemiology and Biostatistics, Saint. Louis University, St. Louis, Missouri, USA; t Research Service, VA St. Louis Healthcare System, St. Louis, Missouri, USA; u Herbert Wertheim School of Public Health & Human Longevity Science, University of California San Diego, La Jolla, CA, USA

**Keywords:** practice effects, repeat testing, serial testing, longitudinal testing, mild cognitive impairment, cognitive change

## Abstract

**Background:**

Repeated cognitive testing can boost performance due to practice effects (PEs), yet it remains unclear whether PEs persist across multiple follow-ups and long durations. We examined PEs across 17 years from midlife to old age in a nonclinical sample.

**Method:**

Men (N=1,608) in the Vietnam Era Twin Study of Aging (VETSA) completed neuropsychological batteries comprising 30 measures across 4 waves over 20 years. We leveraged age-matched replacement participants to estimate PEs at each wave. We compared cognitive trajectories and MCI prevalence using unadjusted versus PE-adjusted scores.

**Result:**

We found significant PEs for multiple tests at all waves, especially in episodic memory and visuospatial domains. Adjusting for PEs resulted in greater cognitive decline and earlier detection of MCI, with up to 20% increased prevalence.

**Conclusion:**

PEs persist across multiple assessments and decades, even with long testing intervals, underscoring the importance of accounting for PEs in longitudinal studies and clinical trials.

## 1. INTRODUCTION

Longitudinal designs are critical for understanding cognitive development and decline [1–4]. In the context of studies on Alzheimer’s disease (AD) and dementia, longitudinal testing is important for identifying risk factors for cognitive decline, understanding variation in disease progression, and evaluating efficacy of treatments [5,6]. However, it has long been acknowledged that performance at follow-up may be artifactually inflated due to practice effects (PEs) [7–9]. Unrecognized PEs obscure the true nature of cognitive decline at later ages with implications for AD and related dementia clinical trials that assess slowing of cognitive decline to determine treatment effects [10–14].

Despite widespread acknowledgment, accounting for PEs is not standard practice in many longitudinal studies. We previously demonstrated that PEs persisted across a 6-year interval and that not accounting for PEs resulted in underdiagnosis of mild cognitive impairment (MCI) and a greater proportion of individuals reverting to normal cognitive status [15]. In an independent sample, we found that adjusting for PEs not only reduced rates of reversion to normal cognitive status, but most importantly, it led to detection of progression to MCI one year earlier. Moreover, practice-adjusted diagnoses were validated based on increased concordance with AD biomarkers [16,17]. We and others have also shown that adjusting for PEs can reduce sample sizes needed in clinical trials, resulting in substantial cost savings [13,16].

PEs are sometimes defined as improved performance at follow-up compared to baseline [18,19]. However, PEs may still exist despite observed decline. In the context of older age or neurodegenerative disease when normative declines are expected (with higher magnitude than PEs), the observed impact of PEs may simply be attenuated decline [20]. One solution proposed by Ronnlund et al. [12] is to utilize attrition replacement approaches that recruit new participants during follow-up study waves who are age-matched to the ongoing longitudinal cohort [see also 15–17,20,21]. By comparing well-matched individuals who differ only on whether they have previously been tested, we can estimate the expected increase in performance due to practice, even when there is an observed decrease in scores at follow-up. This method allows us to create an adjusted follow-up score that can be compared to norms or thresholds for impairment (which assume test scores are driven by current ability and not practice).

Studies of PEs have typically examined test-retest intervals of less than 5 years, with most being in the range of 6 months to 2 years [22,23], and there has been little investigation of PEs in cohorts that have followed individuals for extended periods of time (i.e., over 10 years, but see [10] and [24] for notable exceptions). The Vietnam Era Twin Study of Aging (VETSA) presents a rare opportunity to examine cognitive performance in ~1,600 individuals assessed over 4 study waves and 20 years. We extend the Ronnlund et al. [12] method previously applied to two waves of VETSA data [15], given that the number of assessments has increased with varying patterns of missingness. Here, we apply a generalized approach that leverages AR participants and flexibly handles complex testing schedules to examine how PEs evolve over two decades, and the impact adjusting for these effects has on cognitive trajectories and rates of MCI. We expected that PEs on individual measures would vary by cognitive domain, with greatest effect for tests in the episodic memory domain, and that effects would wane over time. In addition, we compared trajectories of cognitive composite scores and MCI prevalence using unadjusted versus PE-adjusted scores and predicted that adjusting for PEs would result in earlier detection of MCI.

## 2. METHODS

### 2.1. Participants

Participants were 1,608 individuals tested at one or more of four completed study waves of the VETSA. VETSA is an on-going longitudinal study of aging and risk for AD beginning in middle age [25–27]. Participants were members of the Vietnam Era Twin Registry, a national, community-dwelling sample of male-male twins who served during the Vietnam era (1965–1975) [28–30]. All Registry members were invited to participate in the Harvard Drug Study [31], for which ascertainment was not based on any diagnostic or substance use criteria. VETSA participants were then randomly recruited from the Harvard Drug Study sample. At baseline VETSA participants were similar to American men in their age cohort with respect to health, education, and lifestyle characteristics based on Center for Disease Control and Prevention data [32], and nearly 80% reported no combat exposure [30]. Wave 1 data collection occurred between 2003–2007 with follow up data collections at wave 2 (2009–2014), wave 3 (2016–2019), and wave 4 (2021–2024).

**Figure 1.**
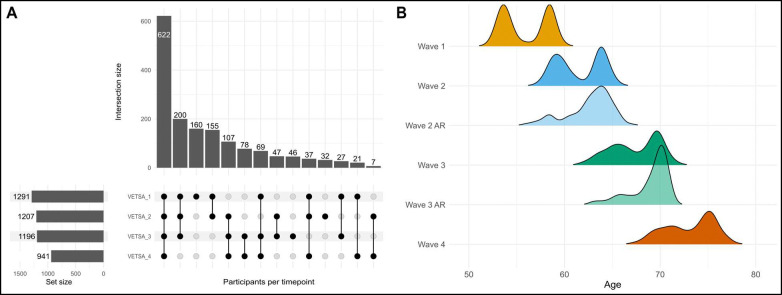
Summary of assessments and age distributions by wave. A) Upset plot describing the patterns of baseline and follow-up assessments completed. Individuals with baseline assessments at waves 2 and 3 were recruited as attrition replacements. B) Density plots of age distributions at each wave. Attrition replacement (AR) participants were recruited at waves 2 and 3 and were age-matched to the on-going longitudinal sample at the corresponding timepoint.

Of the 1,608 individuals in the current study, 1,291 had completed wave 1 baseline assessments. At waves 2 and 3, attrition replacement participants age-matched to the ongoing sample were recruited from the VET Registry and tested for the first time (wave 2 n=193, wave 3 n=124). These participants were then invited for follow-up at all subsequent waves (see Figure 1A for all patterns of assessments). On average, participants were 56 years of age (range 51–61) at wave 1, 62 years (range 56–67) at wave 2, 68 years (range 61–73) at wave 3, and 74 (range 67–79) at wave 4 (see Figure 1B for age distributions by wave). The average time between wave 1 and 2 was 5.7 years, with 5.9 years between waves 2 and 3 and 5.6 years between waves 3 and 4.

Participants traveled to the University of California San Diego (UCSD) or Boston University (BU) to participate in the VETSA. In some cases, when a participant were unable to travel to San Diego or Boston, testers travelled to administer the same testing protocol at a site nearby the participants’ place of residence. The study was performed in accordance with the ethical standards as laid down in the 1964 Declaration of Helsinki and later amendments. Informed consent was obtained from all participants and institutional review boards at both sites approved all study procedures.

### 2.2. Cognitive tests and measures

A set of 30 neuropsychological measures covering multiple cognitive domains were assessed for PEs (see Table 2 for full list of tests and scores by domain). For consistency, scores from tests where lower values indicate better performance (e.g., reaction time) were reverse-coded so that higher scores uniformly indicate better performance. This transformation did not alter the underlying rank order or variance of the original scores and thus had no impact on the shape of the distributions. In addition to the specific abilities, general cognitive ability (GCA) was measured with the validated Armed Forces Qualification Test (AFQT) [33]. The AFQT is a 100-item multiple-choice paper-and-pencil test administered during military induction at average age 20 and was again administered at each VETSA assessment. The AFQT is highly correlated with standard IQ measures (r=.84) [34]. AFQT scores from military induction (hereafter referred to as “age 20 AFQT”) were reported as percentile scores. Therefore, they have been transformed to the standard normal distribution using a probit transform.

### 2.3. Estimation of practice effects

Practice effects were estimated for each of the 30 measures using generalized estimating equations (GEE). We opted for this class of semiparametric regression models because it requires no assumptions about data distributions such as normality to provide valid inferences for virtually all data distributions arising in practice [35]. Let $Y_{\mathrm{iaw}}$ denote the score on a cognitive test measure (dependent variable) and $X_{\mathrm{iaw}}$ the collection of all explanatory (independent) variables available for the *a*^th^ assessment of subject i occurring at wave w*.* As all cognitive outcomes were treated as continuous, the identity link function was used in specifying GEE model [35]:

$$E\left[Y_{\text{iaw}}|X_{\mathrm{iaw}\;}\right]=\beta_{0}+\beta_{\mathrm{age}\;}(\mathrm{age})+\beta_{\mathrm{afqt}\;}(\mathrm{age}\;20\;\mathrm{AFQT})+\beta_{s1}I(s=1)+\beta_{s2}I(s=2)+\beta_{1}I(w=2)\;\;+\beta_{2}I(w=3)+\beta_{3}I(w=4)+\beta_{4}I(w=2\;\&\;a=2)+\beta_{5}I(w=3\;\&\;a=2)\;\;+\beta_{6}I(w=3\;\&\;a=3)+\beta_{7}I(w=4\;\&\;a=3)+\beta_{8}I(w=4\;\&\;a=4)$$

In the GEE specification, no mathematical model is assumed for the (conditional) distribution of $Y_{\mathrm{iaw}}$ given $X_{\mathrm{iaw}}$. Inferences (standard errors and p-values) about the model parameters β are based on a set of estimating equations derived from the above specification. Thus, inferences are valid regardless of how $Y_{\mathrm{iaw}}$ given $X_{\mathrm{iaw}}$ is distributed, so long as the specification above is correct [35].

Wave *w* and Assessment *a* can take values from 1 to 4, with the condition that the value of assessment cannot be greater than the current study wave*.* Additional indicator variables capture the interaction between Wave and Assessment (again, with the condition that *a*≤*w*). Importantly, individuals at each wave can differ on how many previous assessments they have completed. Participants may also miss a study wave but then return for follow-up at subsequent waves. To account for the potential impact of longer intervals between assessments on practice effects, we include the Skip variable *s*. Skip *s* can take the values 1 or 2 to indicate whether they missed 1 or 2 waves prior to the current assessment.

We additionally adjust for age and age 20 AFQT score. The age term allows us to estimate practice effects independent of normative age-related decline. We previously found that the attrition replacement group at wave 2 had significantly lower age 20 AFQT scores compared to the individuals enrolled at wave 1. Given that participants were randomly recruited from the same population (i.e., the VET Registry), this is likely due to random sampling variation. However, differences in “premorbid” cognitive ability between groups may artificially inflate PEs. For example, higher performance in the returnees at follow-up could be due to PEs, but could also be due to their higher average young adult GCA.

The coefficients from the GEE models described above can be interpreted as the expected difference in score on a given measure associated with the relevant wave, assessment number, and whether any prior waves were skipped. Of greatest interest are coefficients β_3_- β_8_, which can be interpreted as PE estimates. That is, the expected difference in performance at a given wave based on having completed one or more prior assessments. We note that the interpretation of β_3_ is somewhat different than β_1_ and β_2_ despite not having an indicator for Assessment *a*>*1*. Because no replacement participants were recruited at wave 4, the reference level corresponds to a second assessment at wave 4 (*w*=*4 & a*=*2)*. Therefore, this coefficient can be interpreted as the PE for individuals who have completed one assessment prior to wave 4 compared to individuals taking the test for the first time, conditional on age. Our primary interest is the patterns of PEs among individuals who completed all 4 assessment waves.

### 2.4. Adjusting test scores for practice effects

For a given individual, we can calculate the expected PE for a test score by summing up the coefficients corresponding to the wave and how many previous assessments they have completed, as well as whether they have skipped any previous assessments. The resulting “adjustment value” is then subtracted from their observed score, resulting in the score that we would expect if they had been taking the test for the first time. As an example, consider an individual who has completed all 4 waves of testing. We can adjust their score on, say, Logical Memory at wave 4 by subtracting the value of β_8_ (i.e., the PE for someone completing a 4^th^ assessment at wave 4). If they had missed wave 3, we would calculate the appropriate adjustment value by adding the estimated effect of skipping one prior assessment (i.e., β_s1_) to the practice effect β_8_. We might expect the adjustment value in this latter case to be smaller, reflecting a reduced PE due to a longer follow-up interval.

### 2.5. Effect of adjusting for practice effects on cognitive factor scores

We have previously shown that composite measures such as cognitive factor scores can improve reliability and prediction of cognitive decline [36]. Therefore, we are interested in determining the extent to which PEs in individual scores impact these composite measures of cognition. We calculated cognitive factor scores across 6 cognitive domains: episodic memory, executive function, verbal fluency, processing speed, visual memory and visuospatial ability. Details of each factor model are described in previous publications [36–39]. The weights used to generate these factor scores were based on previous latent variable analyses of multiple neuropsychological tests. Two versions of each score were created, one using PE-adjusted scores and the other using unadjusted scores. Higher scores reflect better performance in each domain. To test for differences in PE-adjusted versus unadjusted cognitive composite scores at each wave, we used a longitudinal GEE models with an interaction between wave and adjustment (adjusted versus unadjusted).

### 2.6. Effect of adjusting for practice effects on classification of MCI

Classification of MCI was compared both before and after adjusting for PEs. We defined MCI according to the Jak/Bondi approach as described previously [40,41]. To ensure that MCI classification captured cognitive decline rather than lifelong low ability, neuropsychological scores were adjusted using early adult GCA (age 20 AFQT scores) as a covariate. Impairment was defined as having 2+ measures within a domain >1.5 SD below age- and education-adjusted normative means. Individuals with an impaired memory domain were further specified as amnestic MCI (aMCI), and those with impairments in domains other than memory were classified as non-amnestic MCI (naMCI). Differences in the proportion of individuals classified as having MCI at each wave before and after adjusting for PEs were assessed with McNemar’s χ^2^ test.

## 3. RESULTS

### 3.1. Practice effects on cognitive tests across four study waves.

**Figure 2.**
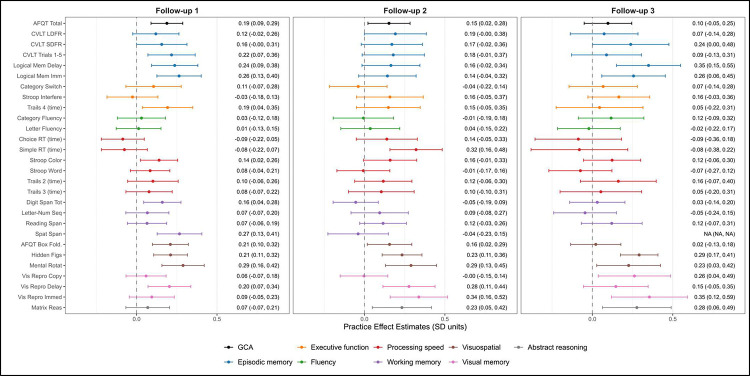
Practice effect estimates across follow-up assessments. The forest plot presents practice effect estimates and 95% confidence intervals at each follow-up assessments (i.e., waves 2, 3 and 4). The presented estimates are for individuals that participated in all four study waves such that estimates reflect practice effects resulting from completing all prior assessments. Dots and whiskers for each measure are colored according to the cognitive domain that they assess. All items were coded such that higher values reflect better performance. See Table 2 for full names of measures in each domain. GCA = general cognitive ability; AFQT = Armed Forces Qualifying Test

We focus on reporting PEs for individuals who attended all four waves. This was the most common pattern of participation and allows us to examine the evolution of PEs over the longest period of follow-up and greatest number of assessments. Figure 2 presents PE estimates and confidence intervals of all measures across each of the three follow-up assessments. At the first follow-up (wave 2), 12 of 30 measures demonstrated significant PEs ranging in magnitude from 0.14 SD units to 0.34 SD units. The largest effects occurred for visuospatial ability and measures verbal episodic and visual memory. At the second follow-up (wave 3), 8 of the 30 measures demonstrated significant PEs ranging from 0.15 SD units to 0.33 SD units. Similar to the first follow-up, practice primarily affected visual memory and visuospatial measures. At the third follow-up (wave 4), 7 of the 30 measures demonstrated significant practice effects ranging from 0.23 SD units to 0.35 SD units. Overall, the number and magnitude of significant practice effects declined slightly over time, though memory and visuospatial tasks consistently showed the strongest effects.

### 3.2. Impact on cognitive factor scores.

**Figure 3.**
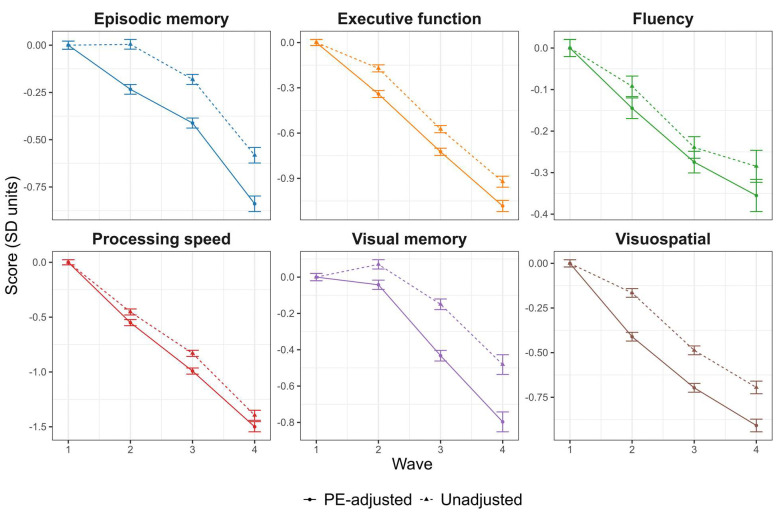
Plots of cognitive factor score trajectories. Means and 95% confidence intervals for cognitive factor score composites calculated from unadjusted (triangles and dashed lines) versus practice effect-adjusted (dots and solid lines) scores. Confidence intervals were calculated using within-subject standard errors. Scores were standardized using the sample means and standard deviations at wave 1.

PE-adjustment led to significantly lower scores across all follow-up waves for all domains (all ps < 0.05), indicating that unadjusted scores may overestimate cognitive performance in these domains. Consistent with results from individual test measures, decreases in performance were most notable in the domains of verbal episodic memory (ranging between −0.24 to −0.26 SD units) and visual memory (ranging between −0.11 to −0.32 SD units), and where weakest in the fluency domain (ranging between −0.04 to −0.07 SD units). See Figure 3 and Supplemental Table S1 for full results of comparisons across waves.

### 3.3. Impact on classification of MCI

**Figure 4.**
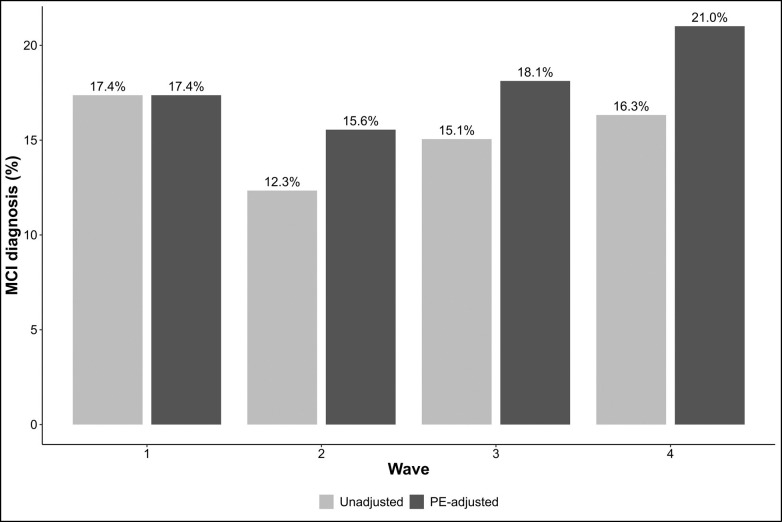
Rates of mild cognitive impairment. Bar plots present prevalence of mild cognitive impairment (both amnestic and non-amnestic) diagnoses based on unadjusted (light grey) and practice effect-adjusted (dark gray) test scores.

We next examined the impact of adjusting for PEs on the rate of MCI at follow-up assessments (Figure 4). Adjusting for PEs significantly increased the estimated prevalence of MCI at wave 2 (12.3% vs. 15.6%, χ^2^=4.821, p = 0.028) and wave 4 (16.3% vs. 20.4%, χ^2^=5.402, p=0.020), with a non-significant upward trend at wave 3 (15.1% vs. 18.1%, χ^2^=3.548, p=0.060). These increases suggest that failure to adjust for practice effects may mask clinically meaningful cognitive decline, particularly in long-term follow-up. Consistent with findings that measures in the memory domains exhibited the strongest PEs, the increased rates of MCI were primarily due to increased prevalence of amnestic MCI. After PE adjustment, the rate of amnestic MCI was significantly higher at all follow-up waves (wave 2: 8% vs 11%, χ^2^=5.213, p=0.022; wave 3: 10% vs 13%, χ^2^=6.333, p=0.012; wave 4: 13% vs 17%, χ^2^=4.546, p=0.033). In contrast, rates of non-amnestic MCI remained stable across waves regardless of adjustment (all ps>0.05), suggesting that practice effects may play a lesser role in these domains.

## 4. DISCUSSION

These results demonstrate the persistence of PEs in certain cognitive measures over extended periods of time and multiple assessments. Our findings are broadly consistent with other studies in that PEs were most apparent at the first follow-up, with weakening – but not absence of – at later follow-ups [19,42–44]. It should be noted that most prior studies finding an that PEs were absent at follow-up examined assessment schedules with much shorter intervals and did not use replacement-participant approaches. Additionally, performance on certain tests may have plateaued at later follow-ups – suggesting no additional PEs – because participants had reached ceiling. Although our 5–6 year testing interval is comparatively long, other studies have also found evidence of PEs after 7 years [10,12,22,45]. Persistent PEs were most notable for memory and visuospatial domains and rates of MCI were up to ~20% higher after adjusting for PEs, driven primarily by increases in amnestic MCI.

One of the few studies to examine PEs for multiple assessments over a comparable period of time found that individuals continued to show practice-related improvements on an intelligence test up to the fourth wave [10,11]. The current study adds additional context by demonstrating the variability in PEs across specific cognitive domains and measures. Consistent with prior studies, we found PEs were most apparent and most persistent in the memory domain, whereas language, attention/working memory, and speed showed the smallest effects [18,19,22,42,46]. This is particularly important given that memory tests such as Logical Memory are used to diagnose amnestic MCI, and our results underscore this point by showing a significant increase in rates of amnestic MCI after PE adjustment. We additionally found strong effects in the visuospatial domain, which is consistent with some studies [19] but not others [22,42]. This likely reflects variability across studies in the specific measures used to assess each domain, as certain tests may be more susceptible to factors such as ceiling effects [42].

An important aspect of the replacement-participants approach used here is that we were able to estimate and adjust for PEs even with observed declines in performance. Although observed increases will occur when the boosts to performance resulting from practice are larger in magnitude than true decline, in cases where actual decline is of larger magnitude the PEs, PEs may serve only to attenuate or obscure accelerated decline [10,20]. This scenario becomes more likely among samples in which greater normative decline is expected such as in older cohorts or those at risk for dementia. Indeed, some studies find a lack of PEs at older ages or in individuals with AD pathology [18,19,43,47]. However, it has been shown that when appropriate methods are used, individuals with MCI and dementia (including prominent memory impairments) do demonstrate benefits from practice [48,49]. We found that by wave 4, adjusting for PEs resulted in over a 20% increase in the rate of MCI, even though most individuals were showing declines in performance. This supports our prior findings that not accounting for PEs delays diagnosis of impairment [17,20]. Delayed detection of MCI has important implications for clinical trials that use conversion to impairment or rates of cognitive worsening as outcomes. Moreover, as shown elsewhere, adjusting for PEs reduces the required sample size resulting in multi-million dollar cost savings and allows for more accurate estimate of treatment effects [13,14,16]. Finally, earlier detection of impairment means that earlier intervention and possible greater slowing of disease progression is possible.

The approach we take in the current study estimates PEs at the group level and applies the same correction to individuals based on their test-retest schedule. Therefore, adjustment only accounts for the average expected boost in performance for a given test. However, several studies have proposed using a lack of PEs at the level of individuals to identify impairment and/or predict risk for AD and future cognitive decline [23]. In such studies, a lack of PEs is often defined as participants that show smaller (or no) increases in performance at follow-up. As we noted, individuals exhibiting performance declines may still be benefitting from practice, and it is not always clear whether relative differences compared to group trajectories reflect lack of practice or simply greater decline. Hence, it will be important for future studies seeking to estimate individual-level PEs to develop robust methods that can disentangle practice effects from decline.

We note some limitations of the current study. The VETSA is an all-male sample and primarily non-Hispanic White, thus limiting its generalizability to other studies. Although PEs were not found to differ by some demographic factors, they have been shown to be influenced by general cognitive ability, age, diagnostic status, and presence of pathology [18,19,21,23,43,47,50–53]. It stands to reason that as the cognitive processes underpinning PE change, so will the resulting boosts in performance. Moreover, study characteristics such as sample age, testing interval, and the specific measures used to assess each domain may impact the magnitude of practice effects [22], and our results provide additional support for this being the case. For these reasons, it is ideal to estimate cohort-specific and test-specific practice effects. In the case of VETSA, recruiting a relatively small number of replacement participants (~10% of the sample) was sufficient to estimate and adjust for PEs with meaningful impacts on measures of cognition and rates of MCI. Our prior results strongly demonstrate that the additional cost of recruiting these participants is outweighed by the benefits, including the potential for substantial cost savings in clinical trials [16]. Importantly, we have also previously implemented an approach that studies without dedicated replacement participants can use to obtain “pseudo-replacements” [16]. These pseudo-replacements then allow for application of replacement-based methods such as those described by Ronnlund et al. [12] or in the current study.

In summary, we found evidence that PEs due to repeated cognitive testing may persist across multiple assessments and over periods as long as 20 years. Significant gains in performance were found even when observed scores declined over time, and this served to obscure the true extent of decline in multiple cognitive domains. Adjusting for PEs had a substantial impact on rates of MCI, with up to a 20% increase in prevalence after adjustment. This meant there was meaningfully earlier detection of progression to MCI. These results underscore the importance of estimating and adjusting for PEs in longitudinal studies of aging and clinical trials aimed at slowing cognitive decline.

## Supplementary Material

Supplement 1

## Figures and Tables

**Table 1.** Demographic characteristics of the sample.

N Total	1608
Age (years); mean (SD)	
*Wave 1*	55.94 (2.43)
*Wave 2*	61.72 (2.45)
*Wave 3*	67.56 (2.53)
*Wave 4*	73.09 (2.09)
Education (years); mean (SD)	13.85 (09)
Age 20 AFQT (percentile); mean (SD)	59.95 (23.11)
Race; n (%)	
*American Indian or Alaskan Native*	5 (0.3%)
*Native Hawaiian or Pacific Islander*	2 (0.1%)
*Black or African-American*	88 (5.5%)
*White*	1492 (92.8%)
*More than one race*	21 (1.3%)
*Decline to answer*	2 (0.1%)
Ethnicity; n (%)	
*Hispanic*	44 (2.7%)
*Non-Hispanic*	1564 (97.3%)
Testing interval (years); mean (SD)	
*Wave 1 to 2*	5.69 (0.69)
*Wave 2 to 3*	5.91 (0.76)
*Wave 3 to 4*	5.63 (0.60)

Note: AFQT = Armed Forces Qualifying Test

**Table 2.** Neuropsychological tests and scores included in practice effects estimation.

Cognitive Domain	Measure
***General Cognitive Ability***	AFQT – Total Score
***Episodic Memory***	CVLT-II Total of Trials 1–5 CVLT-II Short Delay Free Recall CVLT-II Long Delay Free Recall WMS-III Logical Memory - Immediate Recall Story Units Total Score WMS-III Logical Memory - Delayed Recall Story Units Total Score
***Executive function***	D-KEFS Trails 4 time (adjusted for Trails 2 & 3) D-KEFS Category switching accuracy (adjusted for verbal fluency) Stroop: Interference Score (adjusted for color & word) [54]
***Verbal Fluency***	D-KEFS Letter Fluency FAS total correct D-KEFS Category Fluency Animal/Boys total correct
***Processing Speed***	D-KEFS Trails 2 time D-KEFS Trails 3 time Stroop: Raw Word Score [54] Stroop: Raw Color Score [54] Simple Reaction Time Choice Reaction Time
***Short-term/Working Memory***	WMS-III Digit Span: Forward + Backward Score WMS-III Spatial Span: Total Trials Passed Forward + Backward WMS-III Letter-Number Sequencing: Total Score for Trials Passed Reading Span: Total Score Ascending [55]
***Visuospatial***	Mental Rotation: Total Correct Part 1 [56] Gottschaldt Hidden Figures Total Correct All Parts [57] AFQT – Box Folding Subtest WMS-III Visual Reproduction: Copy Total Score
***Visual Memory***	WMS-III Visual Reproduction: Immediate Recall Total Score WMS-III Visual Reproduction: Delayed Recall Total Score
***Abstract Reasoning***	WASI Matrix Reasoning Raw Score

Note: AFQT = Armed Forces Qualifying Test [33]; CVLT = California Verbal Learning Test-II [58]; WMS-III = Wechsler Memory Scale-III [59]; D-KEFS = Delis-Kaplan Executive Function System [60]; WASI = Wechsler Abbreviated Scale of Intelligence [61]

## Data Availability

Instructions for data access requests are available on the VETSA website (https://psychiatry.ucsd.edu/research/programs-centers/vetsa/researchers.html). Access to data from military induction can be requested from the Vietnam Era Twin Registry (https://www.seattle.eric.research.va.gov/VETR/Investigator_Access.asp).
