# Supplementary material for: Practice effects persist over two decades of cognitive testing: Implications for longitudinal research
Source: medRxiv. 2025 Jul 31:2025.06.16.25329587. Originally published 2025 Jun 17. Preprint. [Version 2] doi: 10.1101/2025.06.16.25329587 (PMC12204257; doi:10.1101/2025.06.16.25329587)
Supplement: Supplement 1 [file media-1.pdf]

Supplemental Table S1. Interpretation of GEE model coefficients.

Term	Interpretation
β_{age}, β_{afqt}	The effect of a one unit increase in age or age 20 AFQT score on cognitive outcome, respectively.
β_0	The reference (intercept) will be wave 1 and assessment 1.
β_{s1}, β_{s2}	Effect of skipping one or two waves immediately prior to the current assessment, respectively.
β_1	The expected difference between wave 2 & assessment 1 compared to wave 1 & assessment 1. This can be interpreted as the difference between wave 2 replacement subjects' baseline score and the original samples' baseline score.
β_2	The expected difference between wave 3 & assessment 1 compared to wave 1 & assessment 1. This can be interpreted as the difference between wave 3 replacement subjects' baseline score and the original samples' baseline score.
β_3	The expected difference between wave 4 & assessment 2 compared to wave 1 & assessment 1. Note, this coefficient is interpreted differently than β_1 and β_2 because no participants had their first assessment at wave 4. This can be interpreted as the practice effect for assessment 2 occurring at wave 4.
β_4	The practice effect occurring at wave 2, 2nd assessment.
β_5	The practice effect occurring at wave 3, 2nd assessment.
β_6	The practice effect occurring at wave 3, 3rd assessment.
β_7	The practice effect occurring at wave 4, 3rd assessment.
β_8	The practice effect occurring at wave 4, 4th assessment.

Supplemental Table S2. Effect of practice effect adjustment on cognitive composite scores at follow-up. Longitudinal generalized estimating equations (GEE) were used to examine the difference in performance on cognitive composites calculated from practice effect-adjusted and unadjusted measures. The wave:adjustment interaction terms presented in the table below test whether adjustment results in a significant change in performance at each wave. All scores were standardized using the sample means and standard deviations at wave 1. Therefore, the coefficients can be interpreted as the average difference after practice effect-adjustment in wave 1 standard deviation units.

Domain	Parameter	Coefficient	SE	CI_low	CI_high	p
Episodic memory	WAVE2:Adjustment	-0.24	0.00	-0.24	-0.23	0.00
	WAVE3:Adjustment	-0.23	0.00	-0.24	-0.22	0.00
	WAVE4:Adjustment	-0.26	0.00	-0.26	-0.25	0.00
Executive function	WAVE2:Adjustment	-0.17	0.00	-0.17	-0.17	0.00
	WAVE3:Adjustment	-0.15	0.00	-0.15	-0.15	0.00
	WAVE4:Adjustment	-0.16	0.00	-0.17	-0.15	0.00
Fluency	WAVE2:Adjustment	-0.05	0.00	-0.05	-0.05	0.00
	WAVE3:Adjustment	-0.04	0.00	-0.04	-0.03	0.00
	WAVE4:Adjustment	-0.07	0.00	-0.08	-0.06	0.00
Processing speed	WAVE2:Adjustment	-0.10	0.00	-0.10	-0.09	0.00
	WAVE3:Adjustment	-0.16	0.00	-0.17	-0.16	0.00
	WAVE4:Adjustment	-0.10	0.01	-0.12	-0.09	0.00
Visual memory	WAVE2:Adjustment	-0.11	0.00	-0.11	-0.11	0.00
	WAVE3:Adjustment	-0.28	0.00	-0.29	-0.27	0.00
	WAVE4:Adjustment	-0.32	0.00	-0.32	-0.31	0.00
Visuospatial	WAVE2:Adjustment	-0.25	0.00	-0.25	-0.24	0.00
	WAVE3:Adjustment	-0.21	0.00	-0.22	-0.20	0.00
	WAVE4:Adjustment	-0.21	0.00	-0.22	-0.21	0.00
