# Supplementary material for: Practice effects persist over two decades of cognitive testing: Implications for longitudinal research
Source: medRxiv. 2025 Jun 17:2025.06.16.25329587. Preprint. [Version 1] doi: 10.1101/2025.06.16.25329587 (PMC12204257; doi:10.1101/2025.06.16.25329587)
Supplement: Supplementary file 1 [file media-1.pdf]

Supplemental Table S1. Effect of practice effect adjustment on cognitive composite scores at follow-up. Longitudinal generalized estimating equations (GEE) were used to examine the difference in performance on cognitive composites calculated from practice effect-adjusted and unadjusted measures. The wave:adjustment interaction terms presented in the table below test whether adjustment results in a significant change in performance at each wave. All scores were standardized using the sample means and standard deviations at wave 1. Therefore, the coefficients can be interpreted as the average difference after practice effect-adjustment in wave 1 standard deviation units.

Domain	Parameter	Coefficient	SE	CI_low	CI_high	p
Episodic memory	WAVE2:Adjustment	-0.24	0.00	-0.24	-0.23	0.00
	WAVE3:Adjustment	-0.23	0.00	-0.24	-0.22	0.00
	WAVE4:Adjustment	-0.26	0.00	-0.26	-0.25	0.00
Executive function	WAVE2:Adjustment	-0.17	0.00	-0.17	-0.17	0.00
	WAVE3:Adjustment	-0.15	0.00	-0.15	-0.15	0.00
	WAVE4:Adjustment	-0.16	0.00	-0.17	-0.15	0.00
Fluency	WAVE2:Adjustment	-0.05	0.00	-0.05	-0.05	0.00
	WAVE3:Adjustment	-0.04	0.00	-0.04	-0.03	0.00
	WAVE4:Adjustment	-0.07	0.00	-0.08	-0.06	0.00
Processing speed	WAVE2:Adjustment	-0.10	0.00	-0.10	-0.09	0.00
	WAVE3:Adjustment	-0.16	0.00	-0.17	-0.16	0.00
	WAVE4:Adjustment	-0.10	0.01	-0.12	-0.09	0.00
Visual memory	WAVE2:Adjustment	-0.11	0.00	-0.11	-0.11	0.00
	WAVE3:Adjustment	-0.28	0.00	-0.29	-0.27	0.00
	WAVE4:Adjustment	-0.32	0.00	-0.32	-0.31	0.00
Visuospatial	WAVE2:Adjustment	-0.25	0.00	-0.25	-0.24	0.00
	WAVE3:Adjustment	-0.21	0.00	-0.22	-0.20	0.00
	WAVE4:Adjustment	-0.21	0.00	-0.22	-0.21	0.00
